# Highlighting the potential of *Synechococcus elongatus* PCC 7942 as platform to produce α-linolenic acid through an updated genome-scale metabolic modeling

**DOI:** 10.3389/fmicb.2023.1126030

**Published:** 2023-03-14

**Authors:** María Santos-Merino, Álvaro Gargantilla-Becerra, Fernando de la Cruz, Juan Nogales

**Affiliations:** ^1^Instituto de Biomedicina y Biotecnología de Cantabria, Universidad de Cantabria—CSIC, Santander, Cantabria, Spain; ^2^Department of Systems Biology, Centro Nacional de Biotecnología (CSIC), Madrid, Spain; ^3^Interdisciplinary Platform for Sustainable Plastics towards a Circular Economy-Spanish National Research Council (SusPlast-CSIC), Madrid, Spain

**Keywords:** cyanobacteria, *Synechococcus elongatus* PCC 7942, genome-scale metabolic model, strain-designing algorithms, α-linolenic acid

## Abstract

Cyanobacteria are prokaryotic organisms that capture energy from sunlight using oxygenic photosynthesis and transform CO_2_ into products of interest such as fatty acids. *Synechococcus elongatus* PCC 7942 is a model cyanobacterium efficiently engineered to accumulate high levels of omega-3 fatty acids. However, its exploitation as a microbial cell factory requires a better knowledge of its metabolism, which can be approached by using systems biology tools. To fulfill this objective, we worked out an updated, more comprehensive, and functional genome-scale model of this freshwater cyanobacterium, which was termed *i*MS837. The model includes 837 genes, 887 reactions, and 801 metabolites. When compared with previous models of *S. elongatus* PCC 7942, *i*MS837 is more complete in key physiological and biotechnologically relevant metabolic hubs, such as fatty acid biosynthesis, oxidative phosphorylation, photosynthesis, and transport, among others. *i*MS837 shows high accuracy when predicting growth performance and gene essentiality. The validated model was further used as a test-bed for the assessment of suitable metabolic engineering strategies, yielding superior production of non-native omega-3 fatty acids such as α-linolenic acid (ALA). As previously reported, the computational analysis demonstrated that *fabF* overexpression is a feasible metabolic target to increase ALA production, whereas deletion and overexpression of *fabH* cannot be used for this purpose. Flux scanning based on enforced objective flux, a strain-design algorithm, allowed us to identify not only previously known gene overexpression targets that improve fatty acid synthesis, such as Acetyl-CoA carboxylase and β-ketoacyl-ACP synthase I, but also novel potential targets that might lead to higher ALA yields. Systematic sampling of the metabolic space contained in *i*MS837 identified a set of ten additional knockout metabolic targets that resulted in higher ALA productions. *In silico* simulations under photomixotrophic conditions with acetate or glucose as a carbon source boosted ALA production levels, indicating that photomixotrophic nutritional regimens could be potentially exploited *in vivo* to improve fatty acid production in cyanobacteria. Overall, we show that *i*MS837 is a powerful computational platform that proposes new metabolic engineering strategies to produce biotechnologically relevant compounds, using *S. elongatus* PCC 7942 as non-conventional microbial cell factory.

## 1. Introduction

Cyanobacteria are promising host organisms for the production of compounds with biotechnological applications (Santos-Merino et al., 2019). Their ability to utilize solar energy to fix CO_2_ makes them particularly attractive, especially in an era where the urge of development of sustainable biotechnological processes has gained an increased attention (Rajneesh et al., 2017). As bioproduction platforms, cyanobacteria offer several advantages when compared to plants and algae, such as higher photosynthetic efficiencies (Zahra et al., 2020) and ease of genetic manipulation (Berla et al., 2013). The model cyanobacterium *Synechococcus elongatus* PCC 7942 has been widely explored as a cell factory to produce several value-added compounds, including 2,3-butanediol (Oliver et al., 2013; Nozzi and Atsumi, 2015) and omega-3 fatty acids (Santos-Merino et al., 2018, 2022), among others.

Cyanobacteria are able to naturally produce short-chain omega-3 fatty acids, such as alpha-linolenic acid (ALA) and stearidonic acid (SDA). In the quest to find more sustainable, suitable, and economically viable hosts for the production of omega-3 fatty acids, cyanobacteria, and microalgae have emerged as alternative organisms to native (i.e., fish and plant oils and oleaginous microorganisms) and non-native sources (e.g., genetic engineering organisms) (Galán et al., 2019; Patel et al., 2020). By contrast, cyanobacteria are preferred organisms over microalgae due to their small genomes that generally facilitates manipulation and the availability of a large number of advanced genome editing tools for cyanobacterial genetic engineering (Vavitsas et al., 2021). In the last years, extensive research efforts have been focused on the metabolic engineering of cyanobacterial strains to enhance ALA and SDA production (Dong et al., 2016; Yoshino et al., 2017; Santos-Merino et al., 2018; Poole et al., 2020; Santos-Merino et al., 2022). In many of the cases, the enzymes directly involved in the synthesis of omega-3 fatty acids (i.e., desaturases) have been overexpressed to increase the production yields. There are only a couple of reports where other targets have been exploited to increase omega-3 fatty acids, such as enzymes involved in the saturated fatty acid synthesis (Santos-Merino et al., 2018, 2022) and the vesicle-inducing protein in plastids (Vipp1), a thylakoid membrane formation enhancer (Poole et al., 2020). Limited exploration has been done to identify additional targets in other competitive metabolic pathways with the aim to increase omega-3 fatty acid titers. One of the major obstacles to make omega-3 fatty acid production by engineered cyanobacteria practical and cost-effective is the low productivity levels achieved in these engineered strains (Shinde et al., 2022). Traditional metabolic engineering strategies are the most common avenues used to increase production yields in cyanobacteria. However, since these classical technologies are expensive, time-consuming, and labor-intensive processes, computational biology strategies are emerging as powerful tools to overcome these limitations (Xu C. et al., 2013; Gudmundsson and Nogales, 2021).

Genome-scale models (GEMs) are based on the annotated genome sequence and describe metabolic pathways as stoichiometric coefficients and mass balances of participating metabolites (Gudmundsson and Nogales, 2015). They can be used as computational test-bed to estimate metabolic fluxes using numerical optimization, thus offering a systems-biology tool not only to link genotype to phenotype but also to analyze and contextualize the metabolic capabilities of organisms (Oberhardt et al., 2009). GEMs have been successfully applied to analyze and guide the metabolism of cyanobacteria for production of several target compounds from CO_2_ (Nogales et al., 2013; Santos-Merino et al., 2019; Hendry et al., 2020). To date, three GEMs have been developed for *S. elongatus* PCC 7942: *i*Syf715 (Triana et al., 2014), *i*JB785 (Broddrick et al., 2016), and *i*JB792 (Broddrick et al., 2019). However, these existing models have paid little attention to fatty acid biosynthetic pathways, making it harder to use them as tool to analyze the potential of *S. elongatus* PCC 7942 toward the production of omega-3 fatty acids and related compounds. Then, an updated GEM with a high-quality annotation of fatty acid biosynthetic pathways is urgently required for accurately contextualizing fatty acid metabolism while predicting nutritional, physiological, and genetic scenarios for overproducing omega-3 fatty acids.

Full-facing this challenge, we present here *i*MS837, an updated GEM of *S. elongatus* PCC 7942 using *i*JB792 as a foundation (Broddrick et al., 2019). We validate the accuracy of our GEM using growth performance and gene essentiality predictions. The validated GEM was subsequently used to assess the production of omega-3 fatty, identifying the overexpression of *fabF* as key metabolic target to increase ALA production in agreement with published experimental data (Santos-Merino et al., 2018). Next, we used the model to predict possible engineered metabolic targets to enhance ALA production, identifying a set of ten additional knock-out metabolic targets that resulted in higher yields of this omega-3 fatty acid, albeit not growth-coupled. In addition, mixotrophic conditions using different carbon sources (i.e., glucose and acetate) were evaluated *in silico*, boosting the ALA yields obtained in both cases. These predictions will serve as a starting point for future efforts to design strains and conditions that will potentially improve omega-3 fatty acid production in cyanobacteria.

## 2. Results

### 2.1. Properties of the *i*MS837 GEM metabolic network

**Figure 1:**
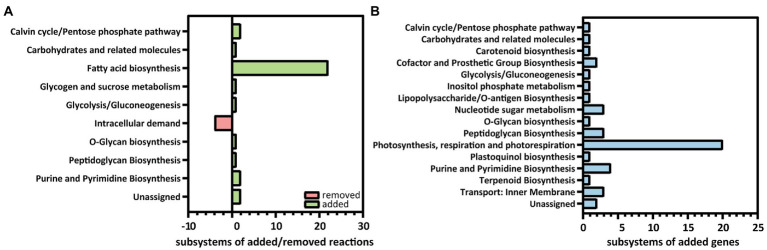
Distribution of reactions and genes added and removed to *i*MS837 based on functional subsystems. **(A)** Summary of reactions added and removed to *i*MS837. **(B)** Summary of genes added to *i*MS837. In both cases, the subsystems where changes have been made are only represented.

*i*MS837 was constructed using as a template the previously published GEM, *i*JB792 (Broddrick et al., 2019). We expanded *i*JB792 by adding new content, including 46 genes, 24 metabolites, and 29 reactions by means of a detailed manual curation based on literature legacy and by comparison with other published high-quality GEMs from cyanobacteria [i.e., *i*JN678 from *Synechocystis*, sp. PCC 6803 (Nogales et al., 2012)] and from heterotrophic bacteria [i.e., *i*JN1462 from *Pseudomonas putida* KT2440 (Nogales et al., 2020)] (Table 1; Supplementary Table S1). Overall, the largest portion of the new metabolic content of *i*MS837 was related with the fatty acid metabolism including 22 new reactions related to this subsystem (Figure 1A). In addition, other 11 reactions were included in *i*MS837, whereas four reactions related to the intracellular demands were removed. These new reactions demonstrate the uniqueness of *i*MS837, especially regarding the modeling of the fatty acid biosynthesis.

**Table 1:** Properties of the different GEM models of *S. elongatus* PCC 7942.

	Values for
	*i*MS837	*i*JB792	*i*JB785	*i*Syf715
Metabolites	801	777	768	838
Reactions	887	858	850	851
Genes	837	791	785	715
Reference	This work	Broddrick et al. (2019)	Broddrick et al. (2016)	Triana et al. (2014)

Beyond the important metabolic expansion done, the gene–protein-reaction (GPR) associations of several reactions were updated while others were corrected in order to improve the model accuracy (Supplementary Dataset 1). For example, reactions involved in glycogen synthesis and degradation were corrected while reactions related to electron transport chain (e.g., Cytochrome *b_6_f* complex, Cytochrome aa3 oxidase, Ferredoxin:NADPH oxidoreductase) were updated, among others. In addition, a meticulous analysis of orphan reactions included in *i*JB792 was carried out in order to identify the genes responsible for such reactions. We manually added 20 new GPRs, which were mainly involved in photosynthesis, respiration, and photorespiration processes (Figure 1B). The rest of the added genes (i.e., 26) belong to a large variety of subsystems as shown in Figure 1B. Finally, the biomass objective function (BOF) of *i*JB792 was also updated in *i*MS837 by removing those metabolites that were not necessary for growth (Supplementary Dataset 1). The modification of the BOF had associated changes in: (i) pigments and xanthophylls, (ii) cofactor pools, and (iii) lipids.

MEMOTE is a platform that has been developed to promote standardization of GEMs, as well as to assess quality control metrics in order to improve model reproducibility and applicability (Lieven et al., 2020). Therefore, we used MEMOTE tool in order to define the completeness, consistency, and interoperability of *i*MS837 when compared with previous models while analyzing potential flaws or shortcomings (Supplementary Dataset 2). The overall score for the model was 75% over the 20% estimated for *i*JB792, which suggest a very good level of completeness. The scores in annotation subcategories were increased by adding annotations and Systems Biology Ontology (SBO) terms to metabolites, reactions, and genes in the updated GEM which were not previously included in *i*JB792. The model scored 55% for the critical category of consistency, which represents accuracy in reaction stoichiometry, mass and charge balances, connectivity of metabolites, and reaction cycles. A major gap was found due the lack of annotation of outside references for some genes, metabolites, and reactions. This limitation only will have some impact when using automated tools or scripts; however, its accuracy and usability should not be affected. Taking together, the MEMOTE analysis demonstrated that *i*MS837 is a highly complete and detailed model that can be used as a reference for other GEM constructions.

### 2.2. Model validation using gene essentiality prediction

**Figure 2:**
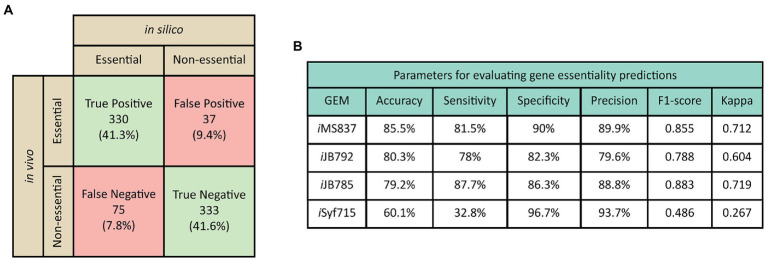
Comparison of gene essentially predictions to experimental results. **(A)** Contingency table of the results obtained for gene essentially predictions using *i*MS837. **(B)** Parameters used to estimate the performance of gene essentially predictions for all GEM of *Synechococcus elongatus* PCC 7942. Gene essentially results obtained *in silico* revealed a high level of accuracy (85.5%), sensitivity (81.5%), specificity (90%) and precision (89.9%) of *i*MS837. Genes with ambiguous essentiality results and not-analyzed *in vivo* were excluded from this analysis (Supplementary Table S2).

An increase in the number of genes, reactions and metabolites does not always indicate a higher-quality GEM. In order to validate the quality of *i*MS837, we conducted an extensive gene essentiality analysis of the genes included in this GEM by comparing the predicted results *in silico* with available essentiality experimental data for *S. elongatus* PCC 7942 (Rubin et al., 2015; Figure 2). We performed single-gene knockout simulations in *i*MS837 using COBRApy (Ebrahim et al., 2013). The model-based gene essentiality predictions showed an overall high-level of accuracy, 85.5% (Figure 2B). More specifically, the model was able to correctly assign 330 and 333 as essential and non-essential genes, respectively (Figure 2A; Supplementary Table S2). On the contrary, the level of discrepancy found between the predictions of *i*MS837 and the experimental data was pretty low. For instance, 75 genes were incorrectly predicted as essential while only 37 genes were predicted as essential but were found non-essential *in vivo*. Gene essentiality accuracy assignment in GEMs is often biased due large number of non-essential genes (Wei et al., 2013). Therefore, to avoid potential bias caused by such effect, we proceed to additionally compute the sensitivity (i.e., proportion of essential genes that have been correctly identified), specificity (i.e., proportion of true negatives that have been correctly predicted), and precision (i.e., the probability that the essential genes were predicted as essential) of our gene essentially prediction (Figure 2B). We found very high values for all these parameters, suggesting no significant bias, and corroborating the high capacity of *i*MS837 when predicting gene essentiality.

On the other hand, when comparing the level of accuracy of *i*MS837 with that from previous *S. elongatus* PCC 7942 GEMs, *i*JB792 (Broddrick et al., 2019), *i*JB785 (Broddrick et al., 2016), and *i*Syf715 (Triana et al., 2014), we found that *i*MS837 scored the highest in terms of accuracy, sensitivity, specificity, and precision (Figure 2B). Whereas *i*JB792, *i*JB785, and *i*Syf715 were able to correctly assign the 80.3, 79.2, and 60.1% of the genes included in the model as true essential and nonessential, the 80.3, 79.2, and 60.1% of the genes included in the model, *i*MS837 outperforms better that these three GEMs by correctly predicting 85.5% (Figure 2B; Supplementary Table S2). Overall, these results indicate that we have not only expanded the last published GEM of *S. elongatus* PCC 7942 by adding new reactions, metabolites and genes, but also, we have improved its accuracy by increasing its completeness.

### 2.3. System evaluation of *S. elongatus* PCC 7942 as a cell factory toward the production of omega-3 fatty acids

The capability of a GEM to provide accurate predictions of experimentally supported data of a target organism’s functional states is a key feature in order to assess the accuracy and completeness of the final reconstruction. Once *i*MS837’s accuracy and completeness were assessed, the model was ready to be used to characterize metabolic states underlying observed phenotypic functions and as a computational framework for metabolic engineering endeavors. In this regard, we further used *i*MS837 as a test-bed to systematically analyze *S. elongatus* PCC 7942 as a cell factory toward the production of ALA. In order to do that, we first included the non-native ALA synthesis pathways in the GEM of *S. elongatus* PCC 7942, a non-natural ALA producer strain. We used *i*JN678, a well-developed GEM from *Synechocystis* sp. PCC 6803 (Nogales et al., 2012) that includes this biosynthetic pathway, since this cyanobacterium is able to naturally produce omega-3 fatty acids (Tasaka et al., 1996). A total of 34 reactions and 28 metabolites were added to *i*MS837 in order to produce ALA and accumulate this omega-3 fatty acid in its membranes (Supplementary Table S3; Supplementary Dataset 1). Among the added reactions to produce ALA in *S. elongatus* PCC 7942, we included the desaturases DesA and DesB (Δ12- and Δ15-desaturases) and all the reactions involved in integrating this omega-3 in the phospholipids of cellular membranes.

*In silico*, the production capabilities of a given strain can be shown using production envelope plots, which represent all possible production rates of a selected metabolite and their associated feasible growth rates (Lewis et al., 2012). Production envelope for ALA production showed that, although feasible under a real nutritional scenario, the production of this omega-3 fatty acid is not coupled to growth (Supplementary Figure S1). In other words, for all the possible levels of ALA production, the model predicted a decrease in the maximal growth rate. This is not surprising since the synthesis of fatty acids is one of the most energetically expensive process among all the lipid membranes components (Zhang and Rock, 2009), and the heterologous production of ALA directly compete with cell growth (Chen et al., 2014). Cyanobacteria only produced unsaturated fatty acids in respond to drops in temperature to compensate for the decrease in membrane fluidity, conditions where they do not normally growth. In addition, it is well-established that cyanobacteria are only able to accumulate fatty acids at low temperatures, where the transcripts for *desA* and *desB* desaturases are more abundant (Sakamoto et al., 1997; Ludwig and Bryant, 2012).

Once demonstrated that the model was able to predict ALA production fluxes, we decided to explore *in silico* previously identified genetic interventions that led to a decrease and an increase in ALA yield (i.e., *fabH* deletion and *fabF* overexpression, respectively) (Santos-Merino et al., 2018). We used Markov chain Monte Carlo sampling to establish potential differences in the metabolic states between strains by comparing the allowed specific metabolic solution spaces (Schellenberger and Palsson, 2009). This flux sampling methodology allowed us to explore the feasible flux solutions in our metabolic network by generating probability distributions of steady-state reaction fluxes (Herrmann et al., 2019). We analyzed the fluxes of the reactions involved in the saturated and unsaturated fatty acid synthesis and their probability in each strain.

**Figure 3:**
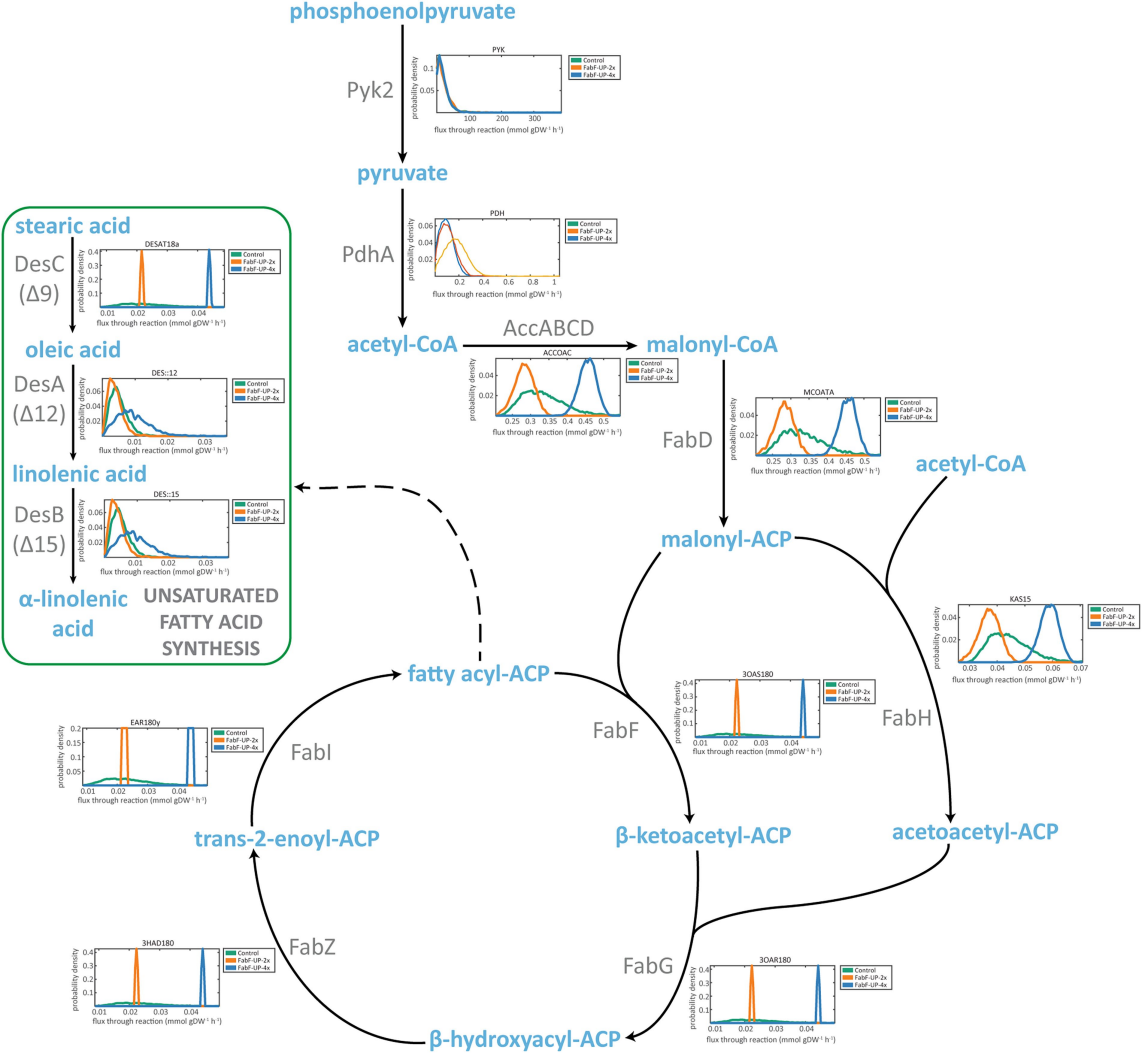
Flux sampling distributions of main reactions of ALA synthesis for *in silico* FabF overexpression mutants and predicted using *i*MS837. For each reaction, a plot of the probability density versus the predicted flux of the specific reaction is represented. In green, the results obtained for *i*MS837_ALA are represented (Control); in orange, an *in silico* designed strain that has double flux for FabF reaction (3OAS180) than *i*MS837_ALA (FabF-UP-2x); and in blue, an *in silico* designed strain that has quadruple flux for FabF reaction (3OAS180) than *i*MS837_ALA (FabF-UP-4x).

Firstly, we explored the flux distributions for strains with increased levels of FabF enzyme (3OAS180 reaction in *i*MS837): (i) FabF-UP-2x, with double flux for FabF reaction than the control strain; and (ii) FabF-UP-4x, with quadruple flux for FabF reaction than the control strain (Figure 3). We observed an overall increase in the flux of reactions involved in the saturated fatty acid synthesis pathway, proportional to the value of FabF flux. Same effect was observed in the flux of the DesC desaturase (DESAT18a). In addition, a large flux for DesA and DesB desaturases (DES::12 and DES::15, respectively) was observed for FabF-UP-4x, but the probability was lower than the minor fluxes observed FabF-UP-2x. These results agree with *in vivo* experimental data linking the overexpression of *fabF* with increased levels of C18:1 (the product of DESAT18a reaction) and ALA (Santos-Merino et al., 2018).

**Figure 4:**
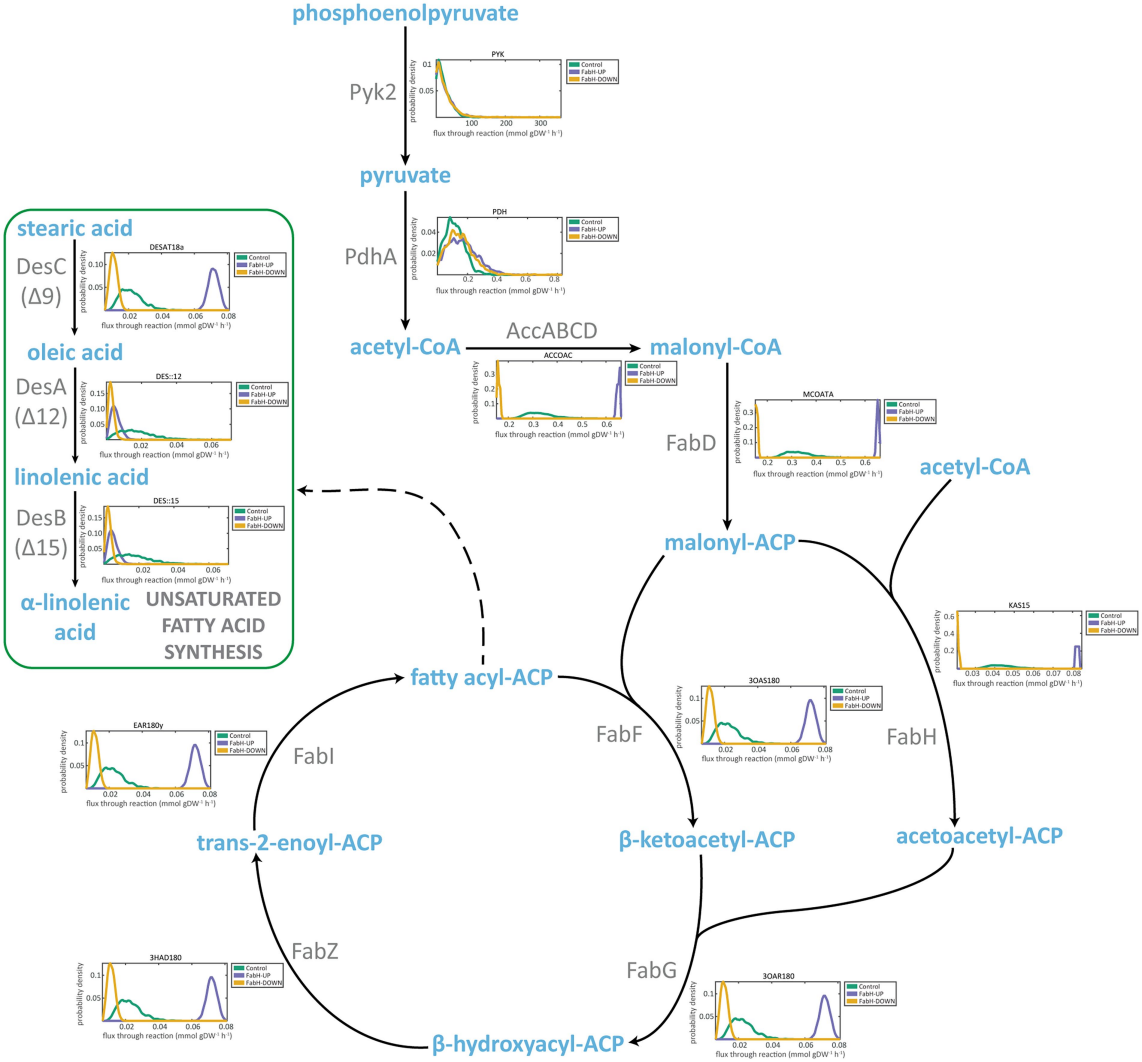
Flux sampling distributions of main reactions of ALA synthesis for *in silico* FabH overexpression and downregulation mutants and predicted using *i*MS837. For each reaction, a plot of the probability density versus the predicted flux of the specific reaction is represented. In green, the results obtained for *i*MS837_ALA are represented (Control); in purple, an *in silico* designed strain that has double flux for FabH reaction (KAS15) than *i*MS837_ALA (FabH-UP); and in yellow, an *in silico* designed strain that has half flux for FabH reaction (KAS15) than *i*MS837_ALA (FabH-DOWN).

As a second scenario, we analyzed *in silico* the flux distributions for strains harboring down- and up-regulation of FabH fluxes (KAS15 reaction in *i*MS837) that we denominated FabH-DOWN and FabH-UP, respectively (Figure 4). For the FabH-UP sampling, it was predicted an increase in the flux of the reactions involved in the elongation cycle (FabF, FabG, FabZ, and FabI). As could be expected, we computed an increase of these fluxes for FabH-DOWN sampling. On the other hand, the flux of DesC desaturase (DESAT18a) was increased in FabH-UP, while it was almost zero for FabH-DOWN. Finally, FabH-UP and FabH-DOWN failed to increase the fluxes through DesA and DesB desaturases (DES::12 and DES::15, respectively). Overall, these results mainly agree with *in vivo* observations, showing no increased in ALA yields after modifications of the expression of *fabH* (Santos-Merino et al., 2018).

### 2.4. Identification of potential gene overexpression targets to increase ALA yields

GEM gives us the advantage of speeding up the exploration and redesign of the metabolism of an organism toward the production of a given metabolite. In most cases, the upregulation of certain fluxes directly or indirectly involved in the biosynthetic pathway of a desired compound, results in improved yields. With the aim of identifying fluxes that could be upregulated to increase ALA production, we used flux scanning based on enforced objective function (FSEOF) (Park et al., 2012). This method scans changes in metabolic fluxes in response to an artificially enforced objective flux of the desired product formation. Using this algorithm, up to 70 target reactions were identified as a result of gradually increments in the ALA production reaction and the acceptance of up to 20% reduction in the biomass-producing reaction (Supplementary Table S4).

**Figure 5:**
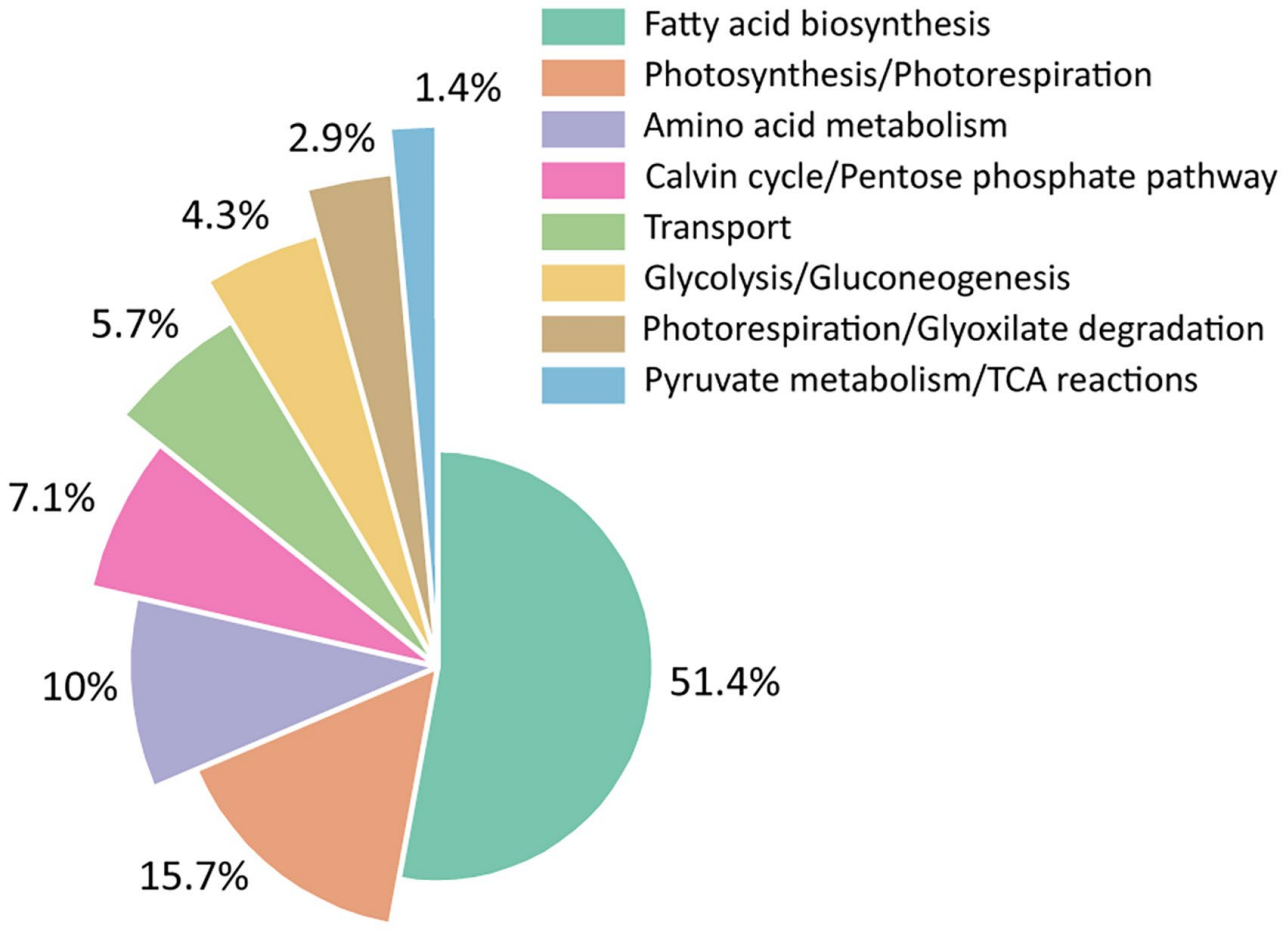
Distribution of reactions obtained with FSEOF algorithm based on functional subsystems. The reactions included in each subsystem are depicted in Supplementary Table S4.

The FSEOF simulation results revealed that ALA production increased with the enhancement of the fatty acid biosynthetic pathways (Figure 5). All the reactions involved in the unsaturation and desaturation steps in the fatty acid biosynthesis were identified as potential overexpression targets. Following the logic of increasing the intermediates of ALA synthesis, we have previously overproduced *in vivo* FabF, FabH, FabD, FabZ, and FabG with the aim to improve ALA yields (Santos-Merino et al., 2018, 2022), with the exception of FabI. The overexpression of most of them did not increase C18:1 levels, the substrate for the sequential activity of DesA and DesB desaturases. Only the overproduction of FabF was able to successfully increase ALA yields. Little known of the regulation of fatty acid synthesis in cyanobacteria, that could affect to our experimental interventions in this pathway, as well as the *in silico* predictive capability of *i*MS837, which does not include information about regulation. Acetyl-CoA carboxylase, which catalyzes the first step of the saturated fatty acid biosynthesis, was also identified as a potential overexpression target to increase ALA production. Overexpression of this enzyme has been proven to be an effective way to increase the rate of saturated fatty acid synthesis in *Synechocystis* sp. PCC 6803 (Eungrasamee et al., 2019), as well as other Acetyl-CoA derived compounds, such as alkanes and alkenes (Tan et al., 2011; Wang et al., 2013). All together indicates that overexpression of Acetyl-CoA could be a feasible strategy to be implemented *in vivo* with the aim to increase ALA yields.

In addition, the increase in the fluxes through the components of the photosynthetic electron transport chain was the second category of reactions that showed an increase in ALA production. It has been experimentally demonstrated that an increase in the light intensity leads to an increase in omega-3 fatty acids in *Synechococcus* sp. PCC 7002 (Sakamoto et al., 1997). Then, it is not surprising that reactions related to photosynthesis are the second-most represented in the results obtained with FSEOF. The third category of reactions that can be upregulated to increase ALA yields is the amino acid metabolism (Figure 5), including reactions involved in the synthesis of alanine, serine, glutamate, glutamine, and aspartate (Supplementary Table S4). In *Arthrospira platensis*, the supplementation of cultures with aspartate stimulated the accumulation of saturated fatty acids, possibly through enhanced *de novo* fatty acid biosynthesis (Fekrat et al., 2022). An increase of the *in silico* flux of reactions associated with amino acid metabolism could have the same role. Finally, it is important to highlight that in the category of reactions related to pyruvate metabolism and tricarboxylic acid cycle (TCA), the only reaction which flux should be increased is the one for the Acetyl-CoA synthetase (Supplementary Table S4). Acetyl-CoA is a key metabolic intermediate that links many metabolic processes, including the TCA cycle, amino acid metabolism, and fatty acid metabolism (Mills et al., 2020). It is possible that in order to increase the direct flux of Acetyl-CoA into the fatty acid synthesis, the flux of this key intermediate should be increased to avoid the competition between all the pathways that use it.

### 2.5. Identification of potential genetic interventions to improve ALA production

In addition to upregulation of reaction fluxes, gene knockout is one the most common strategies to improve microbial strains for producing desirable compounds. OptKnock (Burgard et al., 2003) and GDLS (Lun et al., 2009) are strain design algorithms commonly used to predict genetic manipulations for target overproduction. Both methods are based on constraint-based optimization processes to suggest reaction knockout interventions (constraining the metabolic flux of a reaction to zero) to increase targeted compound production while optimizing biomass yield and product yield. Once identified, the suggested reactions can be eliminated *in vivo* by knocking out one or more of the genes encoding the enzymes catalyzing the reaction.

**Figure 6:**
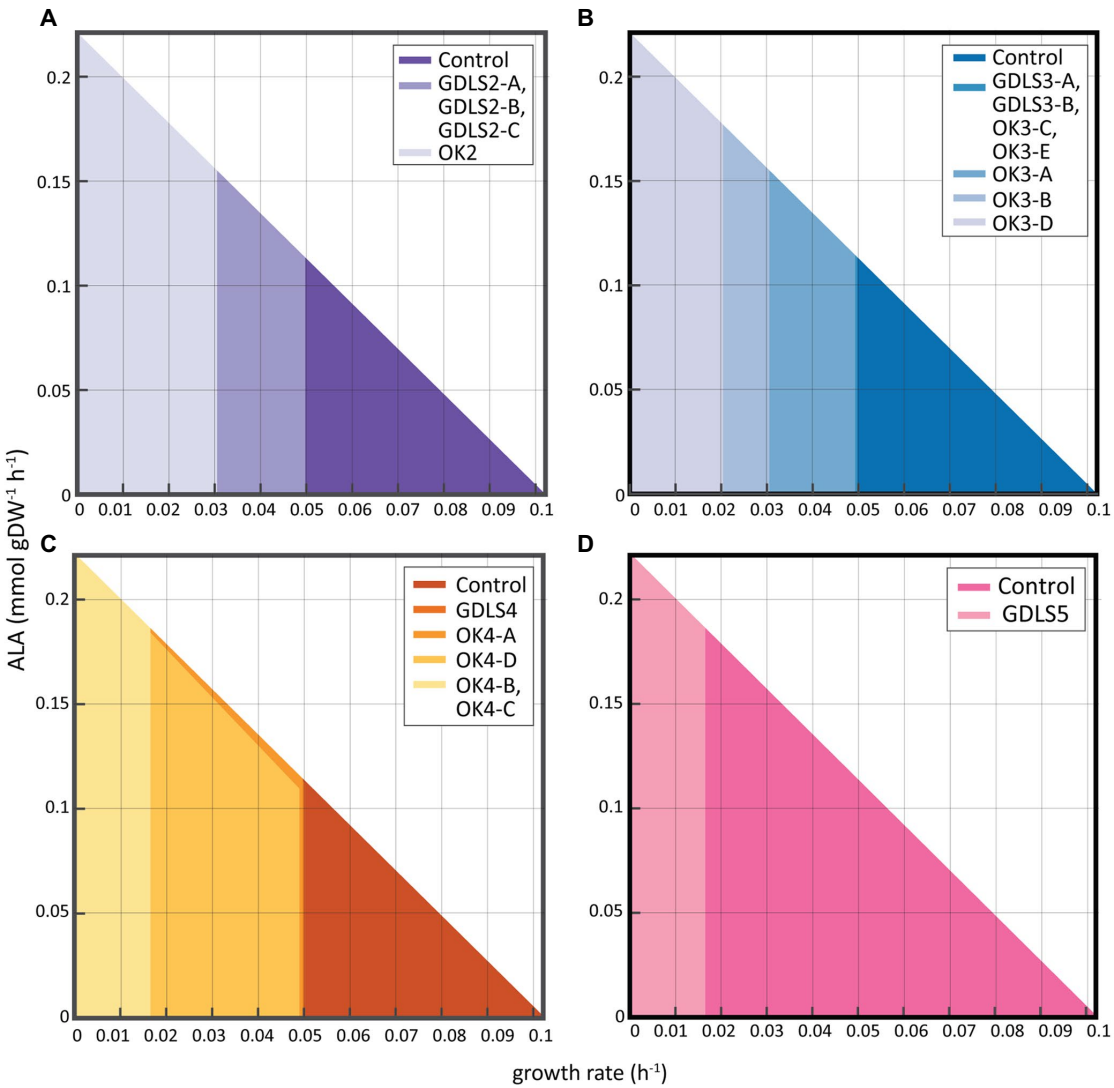
Production envelope for *S. elongatus* PCC 7942 mutants with enhanced ALA production obtained with GDLS and OptKnock. Maximum optimal production rate (mmol gDW^−1^ h^−1^) of ALA achievable with **(A)** two knockouts; **(B)** three knockouts; **(C)** four knockouts; and **(D)** five knockouts.

Unfortunately, the application of GDLS and OptKnock algorithms failed identifying suitable knockout strategies for coupling ALA production to biomass synthesis. However, both algorithms were able to identify partial strategies harboring an increasing number of knockouts putatively resulting in ALA overproduction (Figure 6). Overall, and as could be expected, a higher number of knockouts resulted in a higher ALA production due the removal of competing pathways. Interestingly, Optknock was shown as the most efficient algorithm under the condition tested being able to identify more potential knockout target reactions than GDLS (Supplementary Figure S2; Table 2). Among the six reactions identified as potential target to improve ALA production by both algorithms, four of them were related to amino acid metabolism (ALAD_L, PSERT, PGCD, and PSP_L) and two were involved in the oxidative branch of the Pentose Phosphate Pathway (G6PDH2r and GND, respectively. As mentioned in the previous section, the synthesis of these amino acids outcompetes for Acetyl-CoA necessary for fatty acid biosynthesis. A similar explanation could be attributed to the other two common reactions obtained using OptKnock and GDLS, G6PDH2r and GND, since they are diverting glucose away to the formation of Acetyl-CoA. OptKnock was able to identify a reaction involved in the fatty acid synthesis as a potential target to be knocked out, ACOATA (Supplementary Figure S2; Table 2). This reaction is performed for FabH, an enzyme that failed *in silico* (Figure 4) and experimentally to increase ALA production (Santos-Merino et al., 2018).

**Table 2:** Comparison of the reactions suggested by OptKnock and GDLS to be deleted in order to increase the production of ALA.

Method	Suggested reactions	Enzyme name	Pathway
OptKnock	ACOATA	Acetyl-CoA ACP transacylase	Fatty acid biosynthesis
OptKnock	AGDI	Agmatine deiminase	Amino acid metabolism
GDLS/OptKnock	ALAD_L	L-alanine dehydrogenase	Amino acid metabolism
GDLS	ALCD1	Alcohol dehydrogenase (glycerol)	Glycolysis/Gluconeogenesis
OptKnock	FALGTHLs	Formaldehyde glutathione ligase	Cofactor biosynthesis
OptKnock	FBA	Fructose-bisphosphate aldolase	Glycolysis/Gluconeogenesis
OptKnock	FUM	Fumarase	Amino acid metabolism
GDLS/OptKnock	G6PDH2r	Glucose 6-phosphate dehydrogenase	Calvin cycle/Pentose phosphate pathway
OptKnock	GART	GAR transformylase-T	Purine and Pyrimidine Biosynthesis
OptKnock	GHMT2r	Glycine hydroxymethyltransferase	Amino acid metabolism
GDLS/ OptKnock	GND	Phosphogluconate dehydrogenase	Calvin cycle/Pentose phosphate pathway
OptKnock	H2CO3_NAt_syn	Sodium/bicarbonate symporter (SbtA)	Transport: Inner Membrane
OptKnock	PDS2	Phytofluene dehydrogenase	Carotenoid biosynthesis
GDLS/OptKnock	PGCD	Phosphoglycerate dehydrogenase	Amino acid metabolism
GDLS/OptKnock	PGL	6-phosphogluconolactonase	Calvin cycle/Pentose phosphate pathway
GDLS/OptKnock	PSERT	Phosphoserine transaminase	Amino acid metabolism
GDLS	PSP_L	Phosphoserine phosphatase (L-serine)	Amino acid metabolism
OptKnock	PYK	Pyruvate kinase	Pyruvate metabolism/TCA Reactions
GDLS	VALTA	Valine transaminase	Amino acid metabolism

The optimal solutions for most of the single, double, triple, and quadruple knockouts were different for OptKnock and GDLS predictions (Supplementary Table S5; Figures 6A–C). Only in the case of some triple knockouts, the knockouts in the identified reactions gave identical optimal changes in the flux distribution (Supplementary Table S5; Figure 6B). In addition, only the GDLS algorithm was able to suggest optimal solutions by knocking out five reactions (Supplementary Table S5; Figure 6D). Finally, none of the algorithms was able to provide optimal solutions by applying single reaction knockout strategies.

Although growth coupled overproducing strategies are challenging due the low metabolic robustness of cyanobacteria (Nogales et al., 2013; Gudmundsson and Nogales, 2015), which could result in unfeasible genetic designs under the current scenario, we cannot rule out the possibility that the lack of success using GDLS and OptKnock is due to an insufficiently scrutinized metabolic space. To address a more systematic search, we performed a new strain designing analysis by using gcFront (Legon et al., 2022). gcFront is an algorithm that explores knockout strategies maximizing not only cell growth and product synthesis, but also the strength of production-to-growth coupling using a tri-level optimization. The incorporation of this last optimization parameter significantly reduces the search time with respect other strain designing algorithms such as OptKnock or GDLS, thus significantly speeding up the process. In addition, gcFront is based on a genetic algorithm approach, thus, it allows to perform a larger search in terms of number of knockouts. Unfortunately, we were not able to find growth-coupled ALA overproducing strategies even allowing up to 30 knockouts (data not shown). Taking together, these results confirmed the limited chance of designing growth-coupled ALA overproducing phenotypes using cyanobacteria under photoautotrophic conditions.

### 2.6. Exploring the production of ALA in *S. elongatus* PCC 7942 beyond photoautotrophic conditions

**Figure 7:**
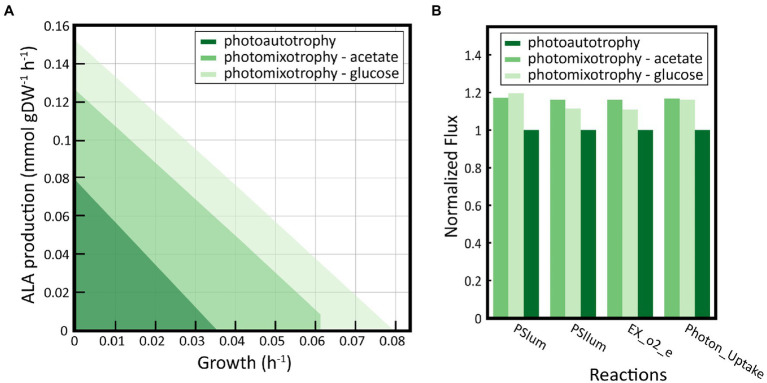
Exploration of the metabolic space under photomixotrophic conditions. **(A)** ALA production envelopes in photoautotrophic and photomixotrophic conditions using acetate or glucose as carbon source. The solution space increases under photomixotrophic conditions. **(B)** Evaluation of photosynthetic activity under photoautotrophic and photomixotrophic conditions. The flux of the reactions associated with the activity of Photosystem I and II (PSIum and PSIIum, respectively), the production of oxygen (EX_o2_e) and the flux oh photons (Photon_uptake) under photomixotrophic were simulated and normalized to the values obtained under photoautotrophic conditions.

The lack of growth coupled ALA designs under photoautotrophic conditions encouraging us to explore alternative nutritional regimens, such as photomixotrophy. It has been previously demonstrated that photomixotrophic conditions can enhance growth performance of *S. elongatus* PCC 7942 while provide, at least in theory, a more robust metabolism increasing the metabolic space suitable to flux rerouting (Yan et al., 2012; Nogales et al., 2013). To address this goal, we constructed a set of condition-specific GEMs using as constraint the uptake of inorganic and organic carbon sources previously reported (Yan et al., 2012), as detailed in methods. Following this procedure, we construct a photoautotrophic model and two photomixotrophic GEMs harboring acetate and glucose consumption systems, respectively. The ALA production envelope of the condition-specific models revealed a significant higher metabolic space bounded by photomixotrophic conditions (Figure 7A). Glucose provided the highest metabolic solution space with up to double production of ALA and growth rate, whereas acetate provided a slightly chance of ALA production although it was not growth-coupled. This improved phenotypic performance under photomixotrophic conditions was not only due the presence of organic carbons as additional nutrients, but also to improved photosynthetic efficiencies (Figure 7B). In fact, we computed significant higher fluxes through Photosystem I and II reactions as well as a higher oxygen evolution and photon uptake under photomixotrophic conditions, completely agreeing experimental data (Yan et al., 2012). Subsequently, we used this expanded metabolic space to search for growth-coupled strategies using gcFront following identical setup than used under autotrophic conditions. Despite several attempts performed, we were not able to identify growth-coupled ALA-overproducing *S. elongatus* PCC 7942 strains neither using glucose nor acetate as organic carbon sources (data not shown). Therefore, we concluded that the single use of knockout strategy is not feasible to reroute carbon flux toward the production of ALA.

To gain further insights on this hypothesis, we analyzed the metabolic flux of the reactions from central metabolism including Calvin–Benson–Bassham (CBB) cycle and TCA and those directly involved in ALA synthesis under photoautotrophic and photomixotrophic conditions (Supplementary Figures S3–S5). The detailed analysis of this flux distribution identified that the synthesis of ALA is limited since Acetyl-CoA pool is funneled almost exclusively to the synthesis of fatty acids. In fact, the metabolism of fatty acids *via* Acetyl-CoA is not connected with the production of other components of the biomass. Compounding the problem, the complete list of genes involved in fatty acid biosynthesis are essential under the three nutritional regimens analyzed, excluding the possibility to reroute carbon flux from fatty acid biosynthesis to ALA production *via* removing competitive pathways. Taking together, the reduced connectivity of Acetyl-CoA and the essentiality of genes surrounding fatty acid biosynthesis and ALA production explains, at great extent, the unfeasibility of designing growth coupled ALA overproducer *S. elongatus* PCC 7942 strains.

**Figure 8:**
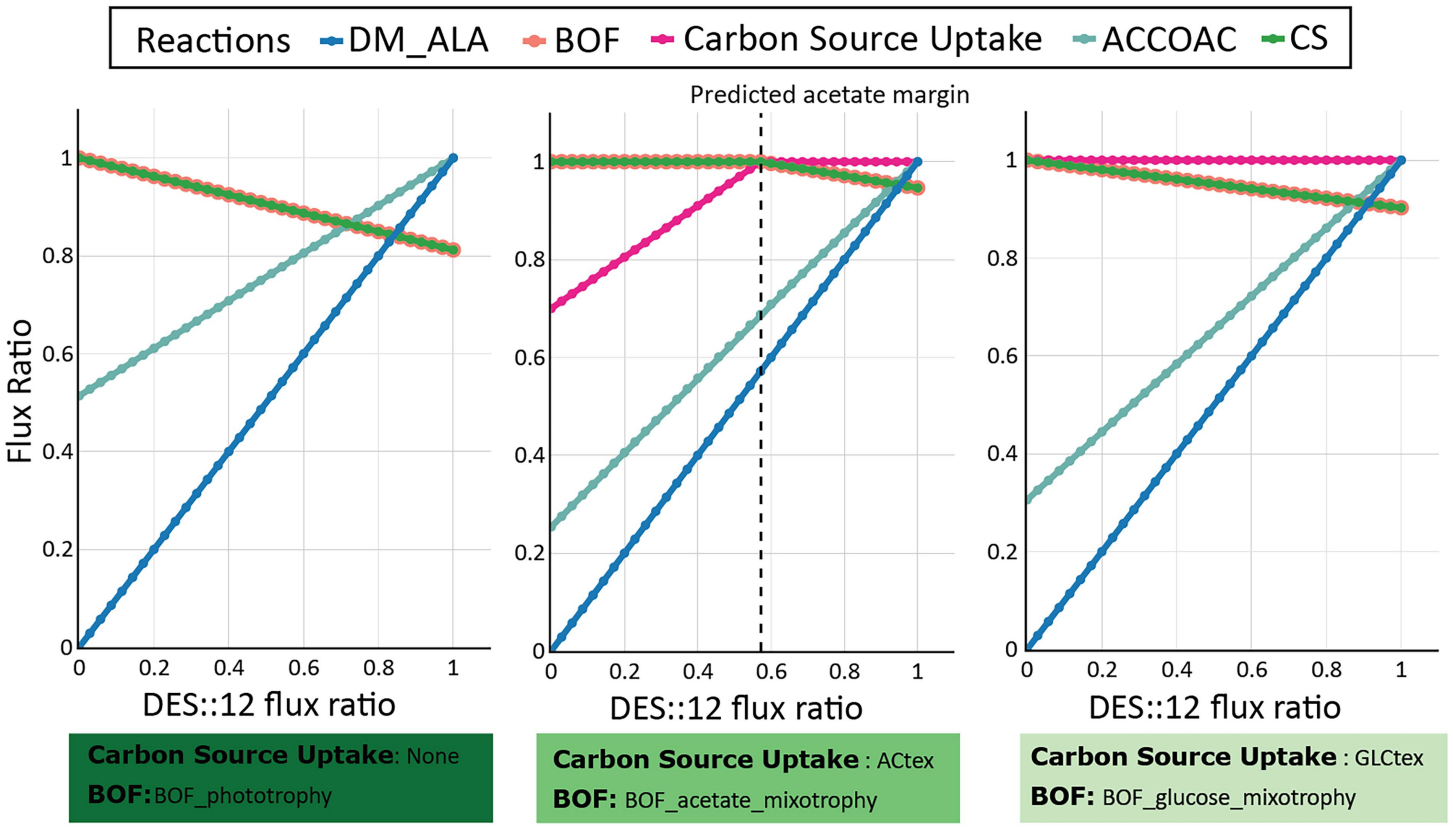
Flux variability analysis of Δ12-desaturase (DES::12) overexpression in different nutritional conditions. Impact of DES::12 overexpression on growth rate (BOF), ALA production (DM_ALA), and organic carbon uptake (Carbon Source Uptake) under photoautotrophic (left panel), photomixotrophic with acetate (middle panel), and photomixotrophic with glucose (right panel) conditions. Fluxes through some reactions of the fatty acid synthesis (ACCOAC) and TCA cycle (CS) were measured.

As a direct consequence, the only solution found increasing ALA production was the increase of the flux through the Δ12-desaturase (DES::12) reaction (Figure 8; Supplementary Figure S3), as it has been demonstrated *in vivo* (Sakamoto et al., 1997; Chen et al., 2014; Santos-Merino et al., 2018). Interestingly, while under photoautotrophic and glucose-driven photomixotrophic conditions the increase in DES::12 flux negatively impacted *S. elongatus* PCC 7942 growth rate (Figure 8; Supplementary Figure S5), we observed an increase in ALA production without any negative effect over the biomass using acetate-driven photomixotrophic conditions (Figure 8; Supplementary Figure S4). This is because, the drainage of the Acetyl-CoA pool toward the ALA production is replenished by increasing the uptake of acetate conditions. Therefore, our computational analysis expanding the metabolic space by feeding *S. elongatus* PCC 7942 with organic carbon sources, strongly suggested that such photomixotrophic conditions, especially using acetate as a carbon source, seem to be a promising strategy to increase ALA production in *S. elongatus* PCC 7942. However, these results will need to be further validated experimentally.

## 3. Discussion

We generated an updated GEM of *S. elongatus* PCC 792 with considerable improvements in model annotation and accuracy of essentiality prediction. The metabolic reaction and genome coverage of the reconstruction was expanded, the format and annotations were updated to be consistent with current best practices. Model improvements were quantified through various metrics such as accuracy of growth yield and ALA yield predictions as well as MEMOTE benchmarking. Overall, *i*MS837 has increased the coverage of the metabolic functionality of *S. elongatus* PCC 7942 and is one of the highest-quality cyanobacterial GEMs. While the updates included in *i*MS837 largely improved the model accuracy, there were still incorrect predictions about gene essentiality that were not able to be addressed. In *S. elongatus* PCC 7942, a significant proportion of genes has still an unknown function (Labella et al., 2020), with many of them presumably involved in processes relevant to the metabolism of the cyanobacteria. It makes it difficult to include new metabolic genes in the model, since there is a lack of information between GPR associations that will allow their identification. In addition, *i*MS837 only contains a small portion of the total genes of *S. elongatus* PCC 7942 genome (837 of 2,772 genes; Table 1) with an absence of regulation that could influence the correct prediction of their essentiality. However, despite all this missing information, *i*MS837 was able to achieve a high overall accuracy for predicting gene essentiality phenotypes (i.e., 85.5%) that is comparable to the performance of the well-curated *E*. *coli* models, *i*JO1366 (i.e., 93.4%) (Orth et al., 2011) and *i*ML1515 (i.e., 93.4%) (Monk et al., 2017).

Although *i*MS837 only captures 30.2% of the protein coding genes of *S. elongatus* PCC 7942, this metabolic reconstruction was able to make correct computational predictions related to experimental data (i.e., gene essentiality and fatty acid biosynthesis). The correct functional annotation of the genes encoding metabolic enzymes involved in the fatty acid biosynthetic pathway helped with prediction involving enzymes of this pathway (Supplementary Dataset 1). An experimental effort to identify unknown GPR associations, as well as a constant improvement and updating of the GEM of *S. elongatus* PCC 7942 will improve its prediction capabilities and its use to generate new hypotheses and to identify promising targets for bioengineering applications (Esvelt and Wang, 2013). Integration of kinetics and omics data in GEMs will broaden their quality and application scopes to better understanding the metabolism of cyanobacteria (Gu et al., 2019). On the other hand, the incorporation of accurate and well-developed GEMs into Design-Build-Test-Learn cycles, together with the use of machine learning, will lead to a very powerful toolset for guiding metabolic engineering of cyanobacteria (Liao et al., 2022).

Metabolic models through the implementation of different algorithms are powerful tools to predict potential interventions that may improve the production of a specific compound. FSEOF and GDLS/OptKnock turned out as efficient systems to predict which gene overexpressions and knockouts, respectively, that might be potential targets to increase ALA production. FSEOF results suggest that the availability of saturated fatty acid pools is important for the synthesis of ALA (Figure 5; Supplementary Table S4). This hypothesis was tested previously *in vivo* demonstrated that FabF seems to be the limiting-rate step in this pathway (Santos-Merino et al., 2018). All the solutions obtained with GDLS, OptKnock and gcFront algorithms failed to produce mutants where ALA production is coupled with growth under photoautotrophic conditions (Figure 6).

It has been previously demonstrated that the carbon flux rerouting to obtain growth-coupled producer strains is more challenging under autotrophic conditions than under mixotrophic or heterotrophic conditions in cyanobacteria (Nogales et al., 2013; Wan et al., 2015). Photomixotrophic culture conditions has been successfully applied in *S. elongatus* PCC 7942 to efficiently increase the production of 2,3-butanediol (McEwen et al., 2013; Kanno et al., 2017), which has been also tested *in silico* using *i*JB792 (Broddrick et al., 2019). In addition, the use of acetate as carbon source has allowed to increase the production of poly-3-hydroxybutyrate (PHB) in different cyanobacterial strains (de Philippis et al., 1992; Wu et al., 2002; Sharma and Mallick, 2005; Panda et al., 2006; Khetkorn et al., 2016; Towijit et al., 2018). Here, we successfully demonstrated that the production of ALA can be also boosted *in silico* under photomixotrophic conditions using acetate or glucose as carbon sources (Figure 7A), and this strategy could be employed *in vivo* likely leading to the same result. The synthesis of PHB and ALA requires the same precursor, Acetyl-CoA. The stimulatory effect of acetate on ALA synthesis could be explained by the direct utilization of acetate to increase the intracellular Acetyl-CoA pool, as it has been previously speculated for PHB (de Philippis et al., 1992; Khetkorn et al., 2016).

Overall, we were able to find potential solutions *in silico* that were feasible to increase ALA production in *S. elongatus* PCC 7942 under phototrophic conditions, but in all the cases, they have a negative cost for the cells, negatively impacting their ability to grow. Omega-3 fatty acid synthesis takes place under a continuous supply of Acetyl-CoA and NADPH, which limits carbon flux through the biomass synthesis (Diao et al., 2020). Using *i*MS837, we were able to predict that there is a limitation of the flux of Acetyl-CoA that impedes maximizing ALA production without decreasing biomass yields (Supplementary Figure S3). Contrary to heterotrophic conditions, phototrophic growth promotes low level of Acetyl-CoA since reducing equivalents are provided by photosynthesis instead of TCA *via* oxidation of Acetyl-CoA. Therefore, Acetyl-CoA is mainly used as building block for fatty acids resulting a narrow window for ALA production (Supplementary Figure S3). The use of an organic carbon substrate to stimulate the production of Acetyl-CoA, such as acetate, was the only alternative option to balance the production of ALA and biomass. The utilization of acetate by *S. elongatus* PCC 7942 does not compete with TCA cycle and CO_2_ fixation activities (Supplementary Figure S4), but also it seems to stimulate *S. elongatus* PCC 7942 photosynthetic activity (Figure 7B), and potentially the NADPH production, needed for fatty acid production.

## 4. Conclusion

In this study, we provided and updated GEM of *S. elongatus* PCC 7942 by using information from the scientific literature and openly available databases, as well as data from well-annotated GEMs from other bacteria. The updated model, *i*MS837, comprised 837 genes, 887 reactions, and 801 metabolites. Following a series of growth simulations, the model was found to agree with published literature. The application of the updated GEM to investigate ALA production recapitulated phenotypes observed in literature, and the use of algorithms to identify potential reactions target to be overexpressed or eliminated can offer systematic strategies that would be difficult to delineate experimentally. In addition, photomixotrophic conditions were also identified as potential target to boost ALA production. This application of the updated reconstruction serves as an example of how GEMs can provide insights into non-intuitive metabolic engineering strategies to improve the production of industrially important metabolites. Ultimately, our computational in-depth analysis of *i*MS837 for ALA production provides an example of the systems-biology science iteration paradigm, by producing further hypothesis that need experimental follow-up to be validated.

## 5. Materials and methods

### 5.1. Development of an upgraded GEM of *S. elongatus* PCC 7942 named *i*MS837

The *i*MS837 model was developed using the published *i*JB792 model (Broddrick et al., 2019) as a starting point (Supplementary Dataset 3). Updates in *i*JB792 were made using Python and the COBRApy package (Ebrahim et al., 2013). A detailed description of all these changes can be found in the Supplementary Dataset 1 and Supplementary Table S1. The new generated model was denominated *i*MS837. All the scripts used in the methods section can be found in GitHub repository (https://github.com/MariaS87/GEM-Synechococcus-elongatus-PCC-7942-iMS837.git).

### 5.2. Flux balance analysis

Flux Balanced Analysis (FBA) uses linear programming to maximize an objective function while assuming no metabolite accumulation during cellular growth. We used FBA to evaluate the biomass production (growth prediction) once the biomass reaction was fixed as the objective function (BOF, Biomass Objective Function) (Orth et al., 2010). The result when executing FBA was the growth rate (h^−1^) predicted under the specified media conditions.

### 5.3. Model manipulation to produce ALA

*i*MS837 was modified to introduce the required reactions to produce ALA. A detailed list of the reactions and metabolites added is included in Supplementary Table S3. The new generated model was denominated *i*MS837_ALA (Supplementary Dataset 3).

### 5.4. Metabolic network simulations

*i*MS837 and *i*MS837_ALA models were analyzed using COBRA Toolbox v2.0 (Schellenberger et al., 2011) within the MATLAB environment (The MathWorks Inc.). Tomlab CPLEX (Tomlab Optimization Inc., San Diego, CA) and Gurobi (Gurobi Optimization Inc., Houston, TX) were used for solving the linear programming problems.

### 5.5. Gene essentiality predictions of *i*MS837

For growth simulation, the biomass equation (BOF) was set as the objective function. The analysis of gene essentiality was performed using the “single_gene_deletion” function of COBRApy (Supplementary Dataset 1; Ebrahim et al., 2013). If the growing rate of the knockout strain was lower than 10^−3^, the gene was defined as essential.

To evaluate the performance of our GEM to correctly predict gene essentiality, we employed a variety of statistical index-based methods, including: accuracy, sensitivity, specificity, precision, F1-score, and Cohen’s Kappa coefficient (van Stralen et al., 2009; Aromolaran et al., 2021). All the statistical metrics were computed based on the scores from true positives (TP), true negatives (TN), false positives (FP), and false negatives (FN). TP and TN occur when both the model prediction and the experimental data agree that a gene is essential and non-essential, respectively. FP occur when the model says a gene is essential, but experiments suggest otherwise, whereas FN occur when the model says a gene is non-essential, but experiments indicate that it is essential (Becker and Palsson, 2008). The aforementioned statistical index-based metrics are described from Equations 1–6 as follows:


(1) $$\%\mathrm{accuracy}=\left(\frac{\mathrm{TP}+\mathrm{TN}}{\mathrm{TP}+\mathrm{TN}+\mathrm{FP}+\mathrm{FN}}\right)\cdot 100$$

(2) $$\%\mathrm{sensitivity}=\left(\frac{\mathrm{TP}}{\mathrm{TP}+\mathrm{FN}}\right)\cdot 100$$

(3) $$\%\mathrm{specificity}=\left(\frac{\mathrm{TN}}{\mathrm{TN}+\mathrm{FP}\;}\right)\cdot 100$$

(4) $$\%\mathrm{precision}=\left(\frac{\mathrm{TP}}{\mathrm{TP}+\mathrm{FP}\;}\right)\cdot 100$$

(5) $$F1-\mathrm{score}=\frac{2\cdot \mathrm{TP}}{2\cdot \mathrm{TP}+\mathrm{FP}+\mathrm{FN}\;}$$

(6) $$\mathrm{Kappa}=\frac{2\cdot \left(\mathrm{TP}\cdot \mathrm{TN}-\mathrm{FN}\cdot \mathrm{FP}\right)\;}{\left(\begin{array}{l} \mathrm{TP}\cdot \mathrm{FN}+\mathrm{TP}\cdot \mathrm{FP}+2\cdot \mathrm{TP}\cdot \mathrm{TN}+\mathrm{FN}\cdot \mathrm{FN}\\ +\mathrm{FN}\cdot \mathrm{TN}+\mathrm{FP}\cdot \mathrm{FP}+\mathrm{FP}\cdot \mathrm{TN} \end{array}\right)\;}$$

The accuracy measures the degree of correctness of a model with respect to both positive and negative classes. The sensitivity estimates the proportion of essential genes that have been correctly identified, whereas the specificity measures the proportion of true negatives that have been correctly predicted. The precision calculates the probability that the essential genes are correctly predicted. The F1-score represents the harmonic mean between precision and sensitivity, combining these two parameters into a single measure (Ghasemian et al., 2022). Lastly, Cohen’s Kappa coefficient measures the degree of agreement between the output of experimental versus predicted essentiality data. If Kappa = 1, then the predictions are in perfect agreement with experimental data, and Kappa = 0 means there is no agreement between predictions and experimental data (Aromolaran et al., 2021).

### 5.6. Monte Carlo flux sampling

The distribution of feasible fluxes in the condition-specific models was calculated by Markov chain Monte Carlo sampling (Schellenberger and Palsson, 2009) implemented in COBRA package (Schellenberger et al., 2011).

### 5.7. Identification of gene overexpression targets for ALA overproduction

The identification of gene amplification targets was based on the strategy of flux scanning based on enforced objective flux (FSEOF) (Choi et al., 2010). We first simulated the growth behavior of the strain using FBA and set the biomass-producing reaction as the objective function. Then, the maximum theoretical ALA production was obtained by setting the ALA exchange reaction (EX_ALA(e)) as the objective function. In the next steps, this reaction was raised stepwise to reach 80% of the theoretical maximum.

### 5.8. Identification of potential knockout targets for ALA overproduction

OptKnock (Burgard et al., 2003) and GDLS (Lun et al., 2009) algorithms were implemented to predict potential genetic knockout manipulations that can lead to ALA overproduction. Whereas OptKnock uses bi-level optimization strategies to solve the conflict of cell growth and maximum bioengineering objective, the GDLS algorithm employs reduced metabolic models and predicts gene knockouts based on Gene–Protein-Reaction associations (Xu Z. et al., 2013). Before using these two algorithms, GEM was reduced, including only nonblocked reactions catalyzed by proteins whose genes are nonessential, reactions not involved in transport and reactions with known GPR associations. This step generated a ‘reduced’ model. These methods were accessible through the COBRA Toolbox v2.0 in MATLAB. For both optimization methods, ALA production flux was set as the optimization target. Each reaction elimination design solution was examined by making the identified changes on bounds to the reactions obtained with OptKnock or GDLS, and were plotted using metabolic production envelope that represents the accessible flux space onto the plane of growth rate versus the target’s production rate (Edwards et al., 2002). The suggested reactions obtained with these algorithms can be removed *in vivo* by knocking out one or more of the genes encoding the enzymes catalyzing the reaction.

In addition, the recently developed gcFront algorithm was also used to identify knockouts that growth-couple synthesis (Legon et al., 2022). gcFront uses a multiobjective genetic algorithm that identifies a Pareto front of designs that maximize growth rate, product synthesis and coupling strength and finds combinations of gene/reaction knockouts that will enforce growth coupling (Legon et al., 2022). Before applying this algorithm, GEMs need to be pre-processed to reduce the search space of reaction by removing the biomass reactions not assigned as objective and blocked reactions. All blocked reactions were identified using flux variability analysis (FVA) (Mahadevan and Schilling, 2003) as reactions unable to carry flux when the biomass was constraint to 20%. To reduce computation time by gcFront algorithm, the list of reactions previously identified with Optknock screening with 1–5 maximum number of knockouts was used. Then, gcFront was executed for each nutritional condition (i.e., phototrophic, photomixotrophic with acetate and photomixotrophic with glucose) using the parameter setup described in Table 3.

**Table 3:** List of parameters used with gcFront algorithm.

Parameters	Values for		
	*i*MS837	*i*MS837_acetate	*i*MS837_glucose
biomassrxn	BOF_photoautotrophy	BOF_acetate_mixotrophy	BOF_glucose_mixotrophy
mingrowth	0.003	0.006	0.008
skipreduction	True	True	True
maxreductionsize	30	30	30
ignorelistrxns	R-C	R-C	R-C
popsize	1,000	1,000	1,000
genlimit	100,000	100,000	100,000

### 5.9. Generation of specific GEMs for photomixotrophic conditions from *i*MS837_ALA

Reactions for the uptake of glucose and acetate were added to *i*MS837_ALA. The experimental data obtained under photomixotrophic conditions with acetate and glucose *in vivo* (Yan et al., 2012) were used to introduce constraints to *i*MS837_ALA to simulate these conditions *in silico*. A summary of these constraints is depicted in Table 4. In addition, a biomass equation was specifically generated for photomixotrophic conditions with acetate and glucose, denominated BOF_acetate_photomixotrophy and BOF_glucose_photomixotrophy, respectively. In order to do that, a previously published protocol to generate biomass objective functions from experimental data was used (Lachance et al., 2019). Condition-specific macromolecular data related to protein, lipid, carbohydrate, and photosynthetic pigment composition were obtained for previously published data (Yan et al., 2012). In addition, transcriptomic data and lipid profiles were also needed and obtained from different literature sources (Rós et al., 2013; Xiong et al., 2015).

**Table 4:** Values of the lower and upper bounds used to generate condition-specific GEMs.

Reactions	Bound values for		
	Photoautotrophy	Acetate photomixotrophy	Glucose photomixotrophy
RBPCcx	(1.46, 1,000)	(1.8, 1,000)	(1.73, 1,000)
ACtex	(0, 1,000)	(0.257, 1,000)	(0, 1,000)
GLCtex	(0, 1,000)	(0, 1,000)	(0.175, 1,000)

### 5.10. Generation of pathway maps for visualization of metabolic fluxes

Escher was used for visualizing the fluxes of the metabolic pathways involved in ALA synthesis (King et al., 2015). The Escher website was used to draw all the metabolic maps.

## Data availability statement

The original contributions presented in the study are included in the article/Supplementary material, further inquiries can be directed to the corresponding authors.

## Author contributions

MS-M and JN conceived and designed the study. MS-M and JN prepared the first draft of the manuscript. MS-M performed the metabolic network reconstruction. MS-M and AG-B performed model simulations. All the authors discussed the results and participated in the writing process.

## Funding

This work was supported by the European Union’s Horizon 2020 Research and Innovation Programme under Grant agreement no. 101000733 (Promicon) by the Spanish Ministry of Science and Innovation (MICINN) grants RobExplode PID2019-108458RB-I00 (AEI/10.13039/501100011033) and TED2021-130689B-C33 to JN, and PID2020-117923GB-I00 to FdlC. Funding was likewise provided by CSIC’s Interdisciplinary Platform for Sustainable Plastics toward a Circular Economy+ (PTI-SusPlast+). MS-M was the recipient of a Ph.D. fellowship (BES-2012-057387) from Spanish Ministry of Economy and Competitiveness (MINECO).

## Conflict of interest

The authors declare that the research was conducted in the absence of any commercial or financial relationships that could be construed as a potential conflict of interest.

## Publisher’s note

All claims expressed in this article are solely those of the authors and do not necessarily represent those of their affiliated organizations, or those of the publisher, the editors and the reviewers. Any product that may be evaluated in this article, or claim that may be made by its manufacturer, is not guaranteed or endorsed by the publisher.
