# Supplementary material for: Highlighting the potential of Synechococcus elongatus PCC 7942 as platform to produce α-linolenic acid through an updated genome-scale metabolic modeling
Source: Front Microbiol. 2023 Mar 14;14:1126030. doi: 10.3389/fmicb.2023.1126030 (PMC10043229; doi:10.3389/fmicb.2023.1126030)
Supplement: Supplementary file 1 [file Data_Sheet_1.DOCX]

Supplementary Material

# Supplementary Data

# Supplementary Dataset S1. Python scripts used for *i*MS837 reconstruction.

# Supplementary Dataset S2. MEMOTE reports for *i*MS837 before and after the manual annotation.

# Supplementary Dataset S3. Files for *i*MS837 and *i*MS837_ALA in JSON format.

# Supplementary Figures and Tables

## Supplementary Figures


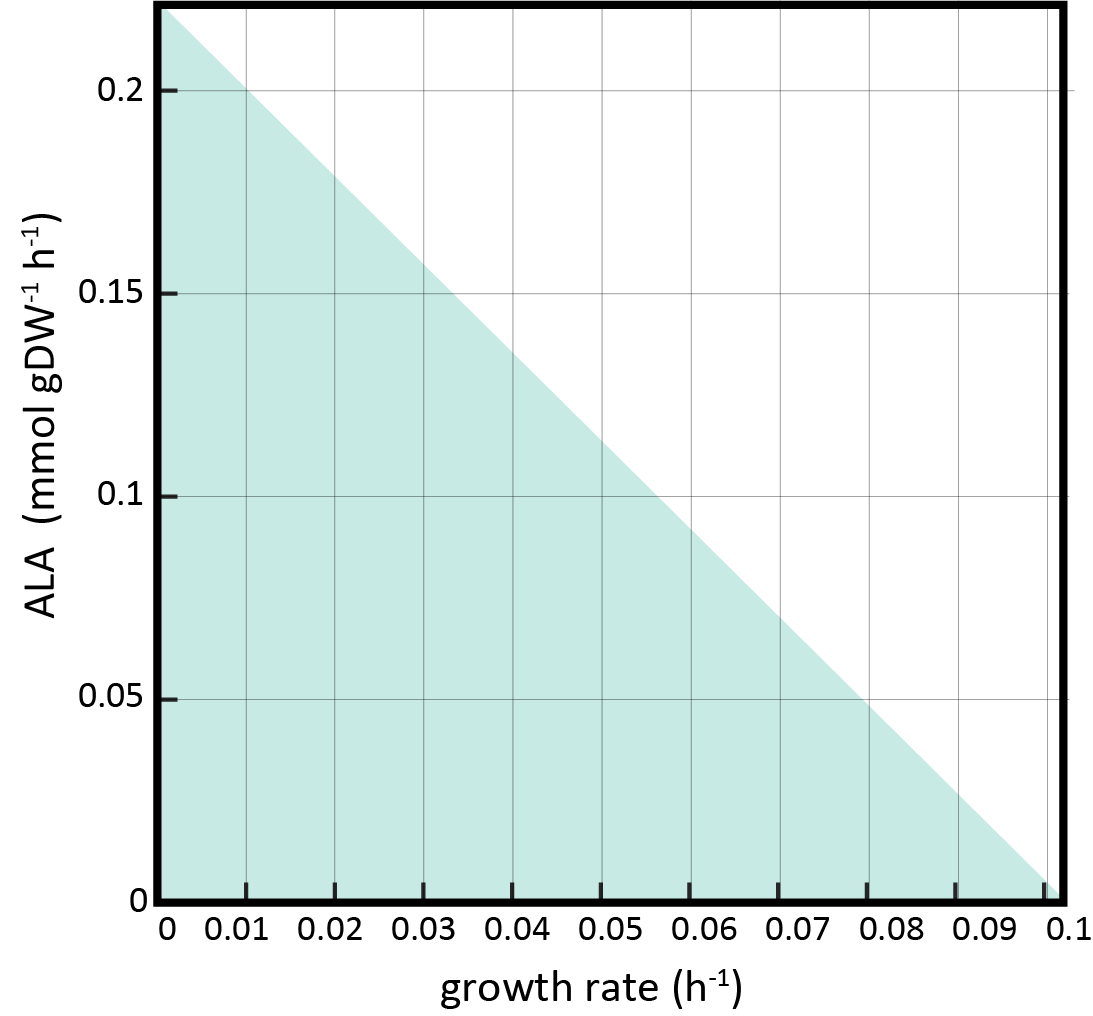


**Supplementary Figure S1.** Production envelope results for optimization of ALA production and biomass formation in *i*MS837.


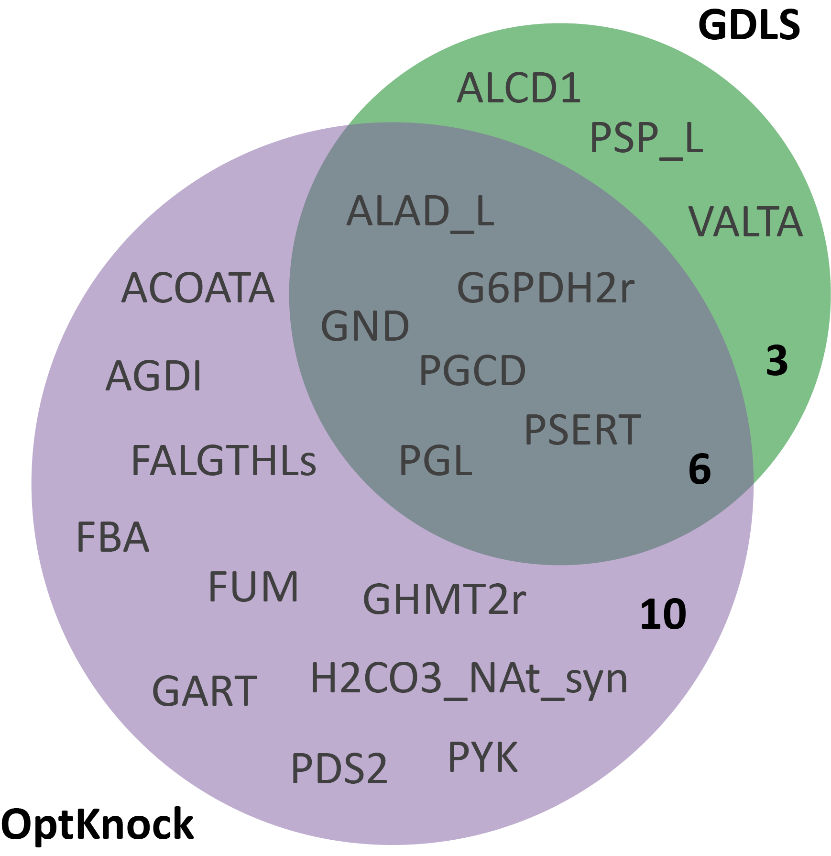


**Supplementary Figure S2.** Venn diagram showing the overlapping reactions to be potentially knocked out to increase ALA production obtained with OptKnock and GDLS algorithms.

**Supplementary Figure S4.**

**Supplementary Figure S5.**


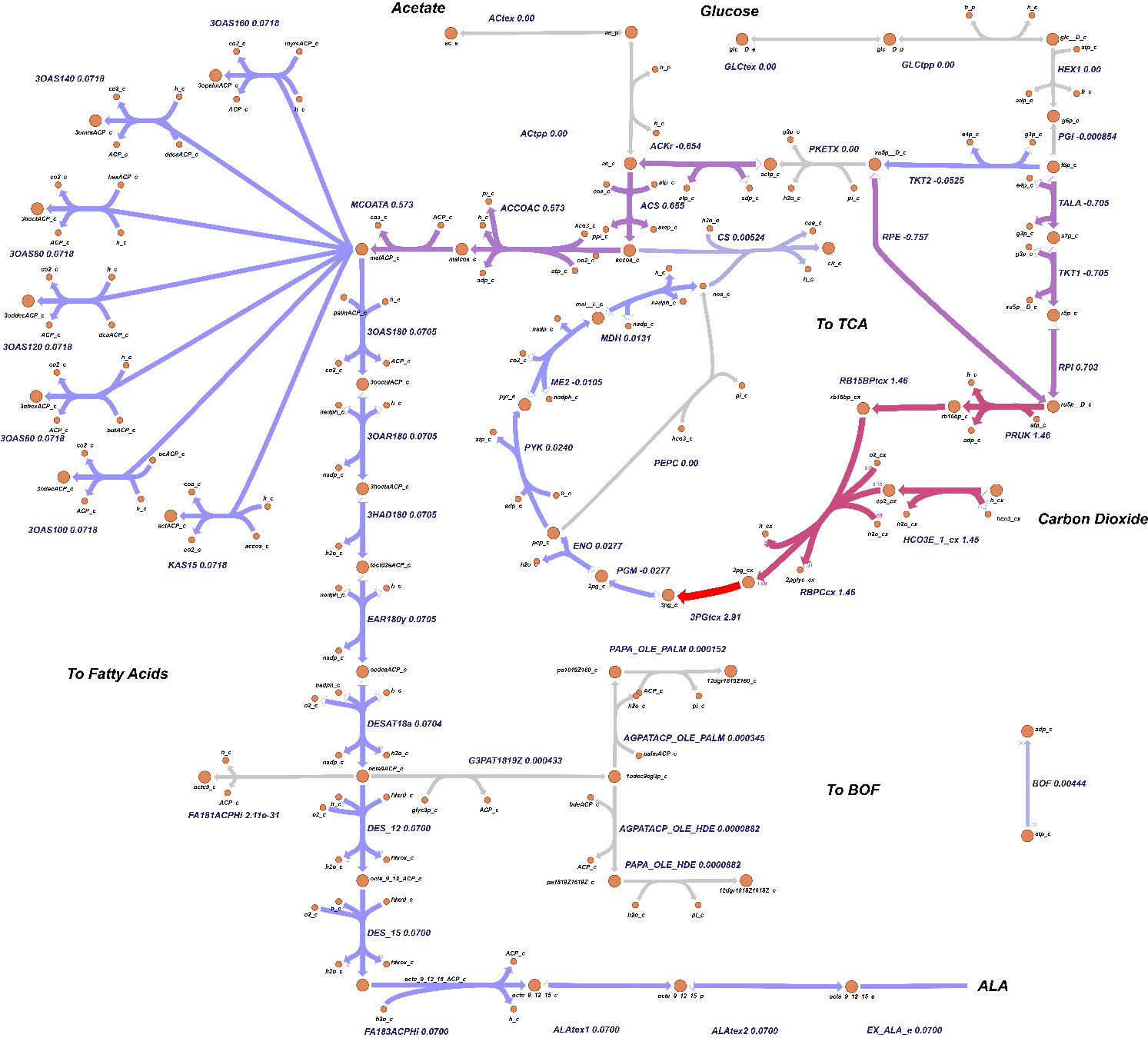
 **Supplementary Figure S3. Predicted distribution of metabolic fluxes in reactions involved in ALA synthesis under photoautotrophic conditions with upregulation of DES::12 (Δ12-desaturase).** The circles refer to metabolites, the connecting lines refer to metabolic reactions, and numbers refer to flux values in mmol gDCW^−1^ h^−1^. Metabolite names are indicated in black, while reaction names are in indicated in blue and red for non-essential and essential reactions in ALA synthesis, respectively. Reaction directionality is represented by arrows. The color of the connecting lines represents the different flux ranges as shown in the legend. Flux values are shown below the reaction abbreviations.


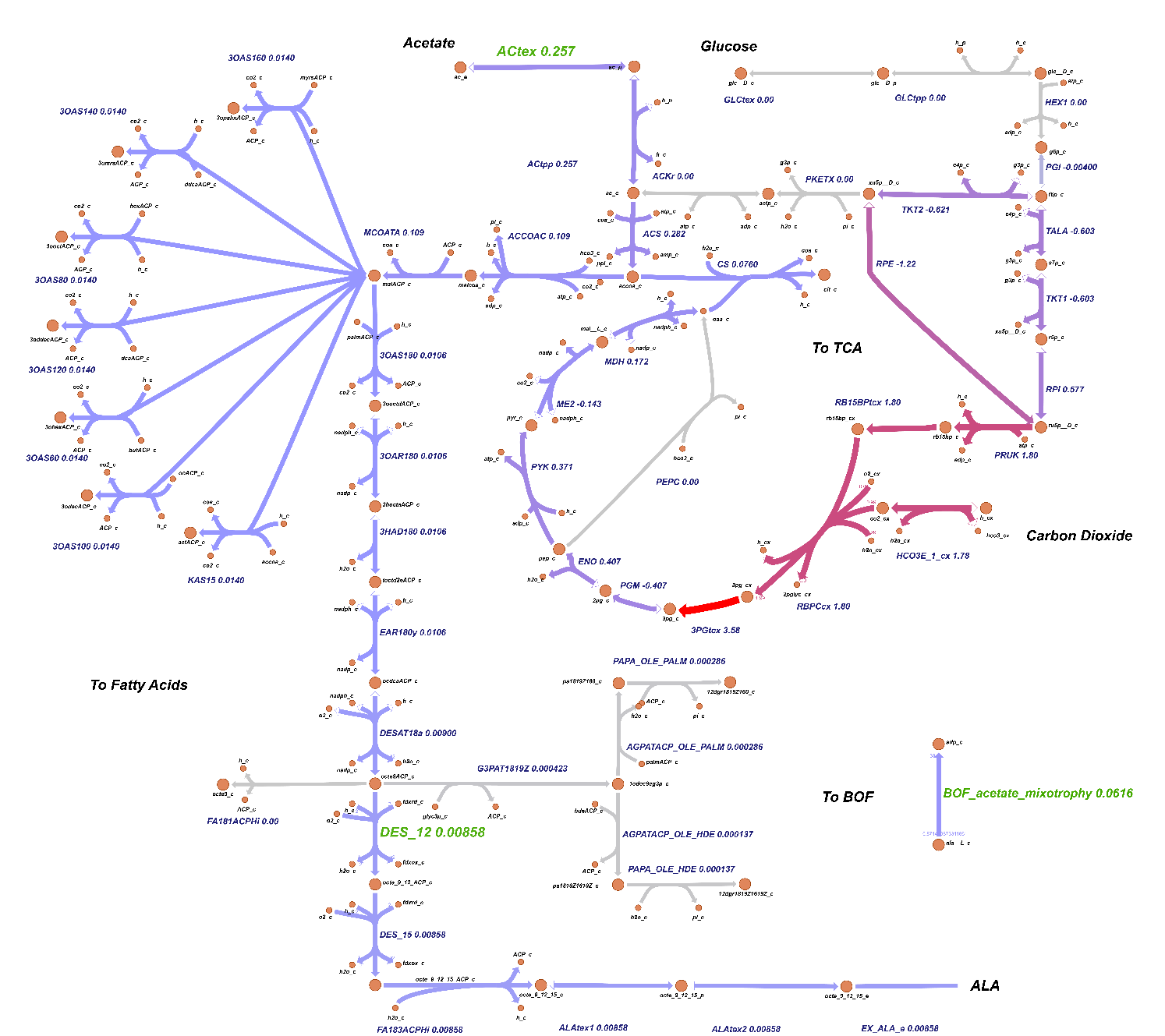


**Supplementary Figure S4. Predicted distribution of metabolic fluxes in reactions involved in ALA synthesis under photomixotrophic conditions with acetate as a carbon source and with upregulation of DES::12 (Δ12-desaturase).** The circles refer to metabolites, the connecting lines refer to metabolic reactions, and numbers refer to flux values in mmol gDCW^−1^ h^−1^. Metabolite names are indicated in black, while reaction names are in indicated in blue and red for non-essential and essential reactions in ALA synthesis, respectively. Reaction directionality is represented by arrows. The color of the connecting lines represents the different flux ranges as shown in the legend. Flux values are shown below the reaction abbreviations.


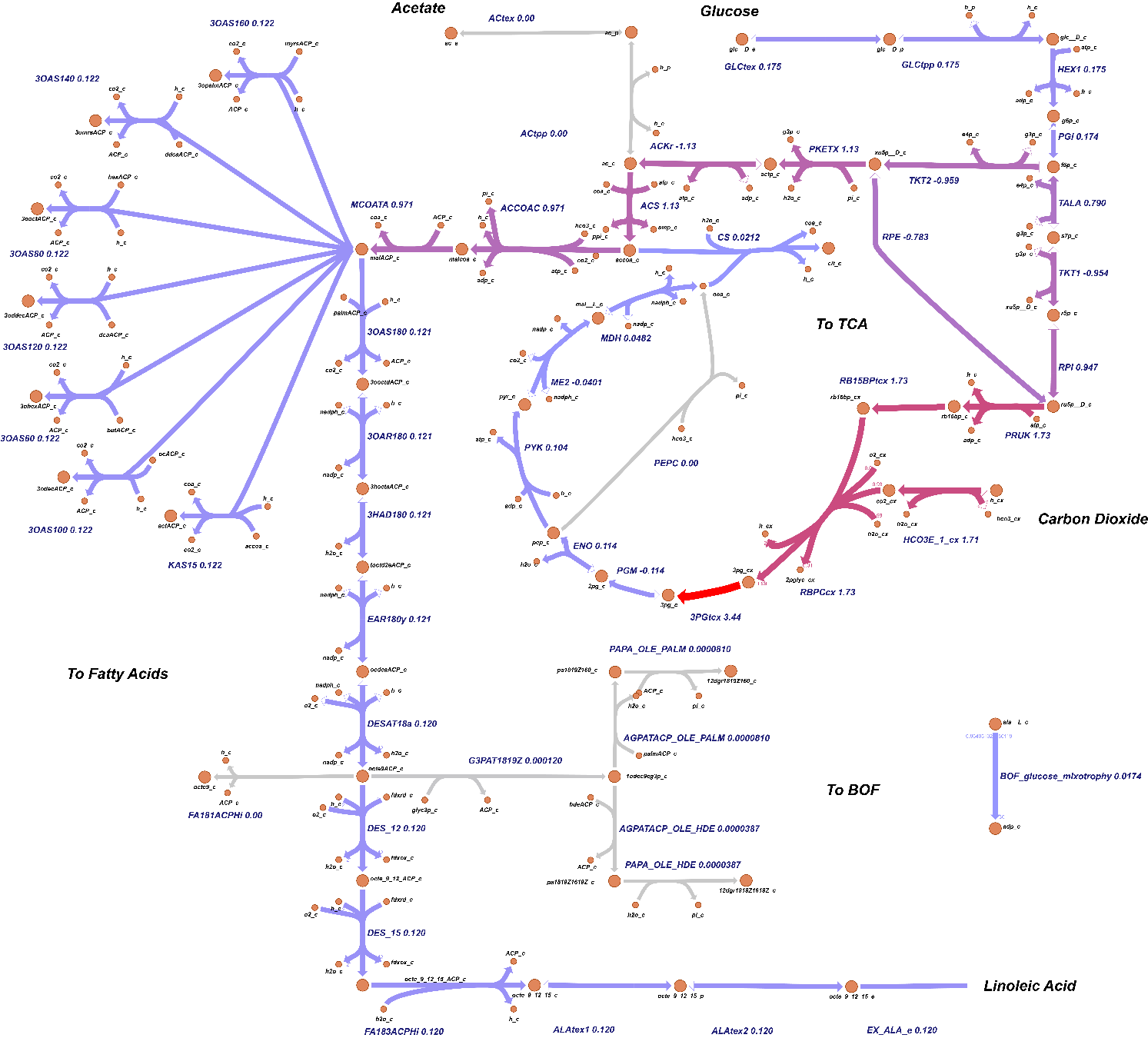


**Supplementary Figure S5. Predicted distribution of metabolic fluxes in reactions involved in ALA synthesis under photomixotrophic conditions with glucose as a carbon source and with upregulation of DES::12 (Δ12-desaturase).** The circles refer to metabolites, the connecting lines refer to metabolic reactions, and numbers refer to flux values in mmol gDCW^−1^ h^−1^. Metabolite names are indicated in black, while reaction names are in indicated in blue and red for non-essential and essential reactions in ALA synthesis, respectively. Reaction directionality is represented by arrows. The color of the connecting lines represents the different flux ranges as shown in the legend. Flux values are shown below the reaction abbreviations.

## Supplementary Tables

**Supplementary Table S1.** List of genes, reactions, and metabolites added and removed to *i*JB792 to generate *i*MS837.

**Supplementary Table S2.** Essential genes predicted using *i*MS837 in comparison with in vivo data.

**Supplementary Table S3.** List of reactions and metabolites added to *i*MS837 to produce ALA.

**Supplementary Table S4.** List of key reactions and genes identified based on the FSEOF simulation.

**Supplementary Table S5.** OptKnock and GDLS determined target reactions with their associated genes.
